# Predictors of Diagnostic Inaccuracy of Detecting Coronary Artery Stenosis by Preprocedural CT Angiography in Patients Prior to Transcatheter Aortic Valve Implantation

**DOI:** 10.3390/diagnostics15060771

**Published:** 2025-03-19

**Authors:** Matthias Renker, Steffen D. Kriechbaum, Stefan Baumann, Christian Tesche, Grigorios Korosoglou, Efstratios I. Charitos, Birgid Gonska, Tim Seidler, Yeong-Hoon Choi, Andreas Rolf, Won-Keun Kim, Samuel T. Sossalla

**Affiliations:** 1 Department of Cardiology, Campus Kerckhoff of the Justus Liebig University Giessen, 61231 Bad Nauheim, Germanya.rolf@kerckhoff-klinik.de (A.R.);; 2 German Centre for Cardiovascular Research (DZHK), Partner Site RheinMain, 60325 Frankfurt am Main, Germany; 3 Department of Cardiology, District Hospital Bergstraße, 64646 Heppenheim, Germany; 4 First Department of Medicine-Cardiology, University Medical Center Mannheim, 68167 Mannheim, Germany; 5 Department of Cardiology, Clinic Augustinum, 81375 Munich, Germany; 6 Department of Cardiology, Munich University Clinic, Ludwig-Maximilians-University, 81377 Munich, Germany; 7 Vascular Medicine & Pneumology, GRN Hospital Weinheim, Cardiology, 69469 Weinheim, Germany; 8 Department of Cardiac Surgery, Campus Kerckhoff of the Justus Liebig University Giessen, 61231 Bad Nauheim, Germany; 9 Department of Cardiology, Justus Liebig University Giessen, 35392 Giessen, Germany

**Keywords:** aortic stenosis, chronic coronary syndrome, coronary artery disease, computed tomography, diagnostic inaccuracy, transcatheter aortic valve implantation

## Abstract

**Background:** The diagnostic performance of preprocedural CT angiography in detecting coronary artery disease (CAD) in patients scheduled for transcatheter aortic valve implantation (TAVI) has been reported. However, data on predictors of diagnostic inaccuracy are sparse. We sought to investigate clinical characteristics and imaging criteria that predict the inaccurate assessment of coronary artery stenosis based on pre-TAVI-CT. **Methods:** The patient- and vessel-level analysis of all CT datasets from 192 patients (mean age 82.1 ± 4.8 years; 63.5% female) without known CAD or severe renal dysfunction was performed retrospectively in a blinded fashion. Significant CAD was defined as a CAD-RADS™ 2.0 category ≥ 4 by CT. Invasive coronary angiography (ICA) served as the reference standard for relevant CAD (≥70% luminal diameter stenosis or fractional flow reserve ≤ 0.80). Pertinent clinical characteristics and imaging criteria of all true-positive (*n* = 71), false-positive (*n* = 30), false-negative (*n* = 4), and true-negative patient-level CT diagnoses (*n* = 87) for relevant stenosis according to ICA were assessed. **Results:** In the univariate per-patient analysis, the following parameters yielded discriminative power (*p* < 0.10) regarding inaccurate CAD assessment by pre-TAVI-CT: age, atrial fibrillation, scanner generation, and image quality. Factors independently associated with CT diagnostic inaccuracy were determined using multivariable logistic regression analysis: a younger age (odds ratio [OR] 0.87; 95% confidence interval [CI] 0.80 to 0.94; *p* < 0.01) and insufficient CT image quality (OR 0.6; CI 0.41 to 0.89; *p* < 0.01). **Conclusions:** Our results demonstrate younger age and poor CT image quality to predict less accurate CAD assessments by pre-TAVI-CT in comparison with ICA. Knowledge of these predictors may aid in more efficient coronary artery interpretations based on pre-TAVI-CT.

## 1. Introduction

Chronic coronary syndromes (CCSs) are evident in up to 81% of patients referred for transcatheter aortic valve implantation (TAVI), a number that is largely affected by age, surgical risk category, and definition of relevant coronary stenosis [1,2]. Due to the high risk of concomitant CCS in patients with severe aortic stenosis (AS), current guidelines primarily recommend invasive coronary angiography (ICA) for the exclusion of relevant coronary artery stenosis [3]. Only on a case-by-case basis is the utilization of computed tomography (CT) angiography selectively supported for this purpose [4].

However, the CT angiography of the aorta and the ilio-femoral access route is indispensable for the preprocedural diagnostic workup of patients with AS prior to TAVI [5]. In each case, TAVI candidates undergo CT angiography in order to obtain information on the severity of AS, the distribution of aortic valve calcium, the aortic annulus dimensions, the distance to the coronary arteries, and the vascular access route [6].

Because of similar protocol requirements of CT planning for TAVI and for coronary CT angiography, the diagnostic yield of pre-TAVI-CT for the assessment of the coronary arteries has been investigated previously [7]. Beyond multiple single-center studies, meta-analyses have investigated the value of pre-TAVI-CT for this purpose [8,9,10,11]. Gatti et al. [11] included data from 14 studies and 2533 patients and reported a pooled sensitivity and specificity of 97% and 68%, respectively. Their results demonstrated a respectable diagnostic accuracy of CT for the diagnosis of significant CCS in TAVI candidates, with the potential to render ICA unnecessary in 41% (95% confidence interval (CI) of 34–47%), assuming a 40% presence of relevant CCS. Within a subanalysis, the use of CT scanners capable of single-heartbeat acquisition was found to improve specificity significantly in comparison with other CT systems (82% vs. 60%, respectively; *p* < 0.01).

However, data on predictors of diagnostic inaccuracy of pre-TAVI-CT are not available [12]. Therefore, the aim of the present study was to add this information to the current literature by assessing clinical characteristics and imaging criteria in order to predict the inaccuracy of detecting coronary artery stenosis based on pre-TAVI-CT.

## 2. Methods

### 2.1. Patient and Study Criteria

In brief, this was a retrospective analysis with the following inclusion criteria: severe symptomatic AS with indication for TAVI and complete diagnostic work-up for TAVI at our institution. Exclusion criteria were as follows: timeframe between CT and ICA > 3 months, previous percutaneous coronary intervention, coronary artery bypass graft surgery, and severe renal dysfunction with an estimated glomerular filtration rate < 30 mL/min/1.73 m^2^. Further details of this study and the patient population were reported previously [13]. This study was performed in accordance with the Declaration of Helsinki and was approved by the local ethics committee. Informed consent was waived because of the study design.

### 2.2. CT Protocol

Pre-TAVI CT evaluation was performed by a 128-slice system (SOMATOM Definition; Siemens Healthineers, Forchheim, Germany) or a 384-slice single-heartbeat scanner (SOMATOM Force; Siemens Healthineers, Forchheim, Germany). Betablockers and nitrates are not part of our routine protocol for pre-TAVI-CT. Nitrates are contraindicated in severe AS, but betablockers may have been administered selectively at the discretion of the responsible physician.

In all patients, a standardized non-contrast Agatston calcium scoring scan in the cranio-caudal scan direction was performed initially. It involved prospective electrocardiographic (ECG) triggering, 120 kV tube voltage, 80 mAs tube current, and diastolic phase reconstruction with 3 mm slice thickness.

The CT angiography scan involved a single bolus of contrast material that was followed by a saline chaser. The cranio-caudal scan was composed of (1) a cardiac acquisition and (2) an aorto-ilio-femoral acquisition. Bolus tracking initiated the acquisition at a threshold of 120 HU in the ascending aorta. A retrospective ECG gating technique was chosen for all CT examinations. For the 128-slice CT angiography, the following parameters were used: 2 × 64 × 0.6 mm collimation, 330 ms gantry rotation time, 110 mL median contrast material, and 120 kV median tube voltage. For the 384-slice CT angiography, the following parameters were used: 2 × 192 × 0.6 mm collimation, 250 ms gantry rotation time, 70 mL median contrast material, and 90 kV median tube voltage.

### 2.3. CT Angiography

The CT datasets were reviewed on an offline workstation (syngo.via; Siemens Healthineers, Forchheim, Germany) by an experienced cardiovascular imaging specialist in a blinded fashion according to society recommendations [14]. Diastolic and systolic cardiac phases were routinely reconstructed using a standard vascular kernel and an additional sharper kernel, as appropriate. A CAD-RADS™ (Coronary Artery Disease Reporting and Data System) 2.0 category ≥ 4 was considered to indicate the presence of relevant coronary artery stenosis here [15]. While category 4a is known as the presence of ≥70% coronary artery stenosis in any coronary artery, category 4b involves ≥ 50% stenosis of the left main artery or triple vessel disease with ≥70% stenosis, and category 5 involves the presence of total coronary artery occlusion. The assessment of coronary artery evaluability and the localization of lesions was based on a 15-segment model. The definitions of the signal-to-noise ratio and image quality were detailed previously, with intra- and inter-reader validation reaching a good correlation [13].

### 2.4. Invasive Reference Standard

ICA was performed in adherence to an institutional protocol and served as the standard of reference. Based on the guidelines, coronary artery stenosis ≥ 70% per visual assessment was defined to indicate hemodynamic relevance [3]. Fractional flow reserve (FFR) was performed to further interrogate an intermediate coronary artery stenosis of 50–70% per local protocol in 15 patients. Seven cases were finally classified as hemodynamically relevant based on FFR values ≤ 0.80.

### 2.5. Statistical Analysis

The assessment for normal distribution was performed graphically and by the use of the Shapiro–Wilk test. The mean ± standard deviation or median [interquartile range, IQR] denote continuous variables, as appropriate. Total numbers with frequency or percentage indicate categorical variables. For normally and non-normally distributed data, the two-tailed unpaired Student’s *t*-test and Mann–Whitney U-test were used, respectively. For categorical variables, the association with the diagnostic concordance of CT angiography and the invasive reference standard was analyzed using the Chi-squared test. Potential confounders yielding *p*-values < 0.1 in the univariate analysis were included in the multivariable regression models. Supplementally, variables with *p*-values < 0.2 were included.

Two-sided *p*-values < 0.05 were defined as being statistically significant. All analyses were performed using dedicated software (MedCalc version 23.0.5 for Windows; MedCalc, Ostend, Belgium).

## 3. Results

### 3.1. Patient and Vessel Characteristics

A total of 192 patients (122 female; mean age 82.1 ± 4.8 years) with 576 coronary vessels were included in the final analysis. The Agatston score of 489.3 [173.6–1203.4] reflected severe calcification of the coronary arteries. Based on the invasive reference standard, relevant CAD was present in 75 (39.6%) patients and 127 (22.0%) vessels. At the patient level, the CT diagnosis was true-positive (TP), false-positive (FP), false-negative (FN) and true-negative (TN) in seventy-one, thirty, four, and eighty-seven patients, respectively. At the vessel level, the CT diagnosis was true-positive (TP), false-positive (FP), false-negative (FN) and true-negative (TN) in 115, 60, 10, and 391 vessels, respectively. Therefore, the diagnostic concordance between pre-TAVI-CT and the invasive reference standard was present in 158 patients (82.3%) and 506 (87.8%) vessels, whereas discordance was observed in the remaining 34 (17.7%) patients and 70 (12.2%) vessels.

### 3.2. Univariate Analyses

The results of the univariate analyses for the evaluation of relevant clinical and imaging covariates to predict the patient-level discordance of pre-TAVI-CT and the invasive reference standard are provided in Table 1. Vessel and lesion characteristics on the pre-TAVI-CT according to vessel-level diagnostic discordance between CT and the invasive reference for the overall study population are shown in Table 2. In addition, Supplementary Table S1 (patient level) and Supplementary Table S2 (vessel level) compare the results separately for FP and FN results. Variables yielding *p* < 0.1 for patient-level discordance in the diagnosis of relevant CAD among pre-TAVI-CT and invasive coronary angiography were patient age, atrial fibrillation, CT system, and CT image quality.

### 3.3. Multivariate Logistic Regression Analysis

**Figure 1:**
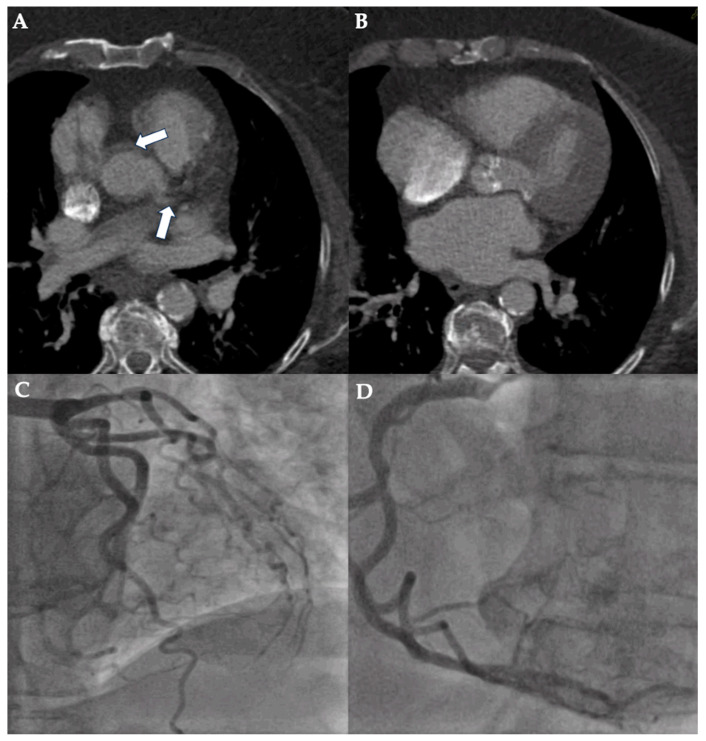
Case example of a false-positive assessment of pre-TAVI-CT. CT planning for TAVI of a 74-year-old woman with body mass index of 43.9 kg/m^2^ was performed using first-generation dual-source CT. Poor image quality (non-diagnostic; 5-point Likert scale rating: 1) due to the combination of excess quantum mottle, stairstep artifact (white arrows show double contour of aorta and left main coronary artery), and suboptimal coronary artery contrast is illustrated in axial image views (**A**,**B**). However, ICA excluded relevant coronary artery disease of the left (**C**) and right coronary artery (**D**).

**Figure 2:**
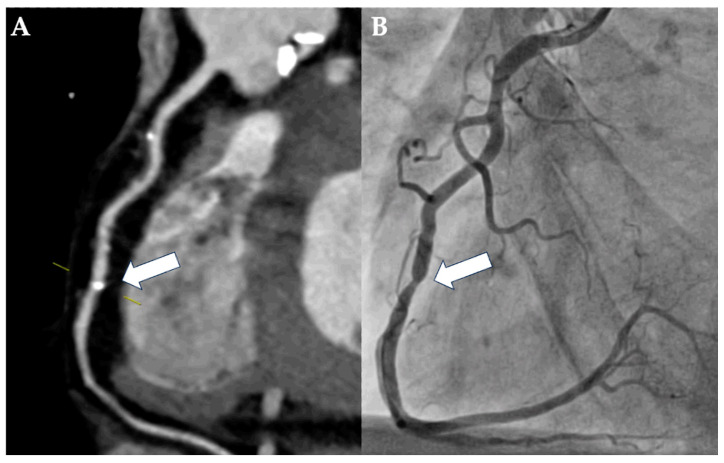
Case example of a rare false-negative assessment of pre-TAVI-CT. A 58-year-old man with severe AS was evaluated for TAVI due to hostile chest and chronic obstructive pulmonary disease with long-term oxygen therapy. Imaging was performed using first-generation dual-source CT and was of good quality despite minor streak artifacts arising from contrast material in the right ventricle. Based on the vessel view of the right coronary artery (**A**), the localized calcification of the mid segment was assessed as being mild (25–49% luminal diameter stenosis; CAD-RADS 2; white arrow). However, ICA revealed a severe, ca. 80% luminal diameter stenosis of the corresponding lesion in the mid-segment of the right coronary artery ((**B**); white arrow).

The results of the multivariate logistic regression analysis for the evaluation of diagnostic inaccuracy regarding CAD by CT prior to TAVI and invasive coronary angiography are demonstrated in Table 3 (patient level) and Table 4 (vessel level). In addition, Supplementary Table S3 (patient level) and Supplementary Table S4 (vessel level) show the results of the multivariate logistic regression analysis for all variables yielding *p* < 0.2 in the univariate testing, in order to rule out that other relevant variables (i.e., body mass index, heart rate and hypertension on a per vessel level, and vessel-specific signal-to-noise ratio on a per vessel level), may have had a confounding effect on the results. A younger patient age (odds ratio (OR), 0.87; CI 0.80–0.94; *p* < 0.01) and worse CT image quality (OR, 0.60; CI 0.41–0.89; *p* < 0.01) were both independently associated with patient-level CT diagnostic discordance compared with ICA. At the vessel level, none of the variables showed independent predictive power. Representative patient examples are provided (Figure 1 and Figure 2).

## 4. Discussion

### 4.1. Major Findings

The present study identifies key clinical and imaging predictors of diagnostic inaccuracy in the detection of coronary artery stenosis using preprocedural CT angiography in patients undergoing TAVI. By analyzing patient and imaging characteristics, we provide insights into factors that influence the reliability of CT angiography in this specific population, emphasizing the importance of optimizing strategies to improve their outcomes.

Our findings show that a younger patient age and insufficient CT image quality may have a significant impact on diagnostic inaccuracies, as these were independent predictors of erroneous coronary artery assessment with pre-TAVI-CT on a per-patient level. In this context, it is important to mention that supplementary analyses (Supplementary Tables S3 and S4) were performed in order to rule out that other relevant variables had an effect on the presented results. Nevertheless, a younger patient age and image quality persisted as independent predictors of an inaccurate coronary artery assessment by pre-TAVI-CT.

The association between a younger age and misdiagnosis may be attributable to differences in coronary anatomy, vascular calcification patterns, or the prevalence of soft plaques that are less easily visualized on CT. Furthermore, we believe that a younger patient age in this collective of patients was associated with CAD misdiagnosis on pre-TAVI-CT because of an increased risk of expectation bias and a more focal rather than extensive disease of the coronary arteries. This suggests that closer attention must be given to optimizing imaging settings in younger patients with severe AS who are referred for TAVI, and supplementary diagnostic modalities, such as ICA, should be considered.

Poor image quality often results from motion artifacts, severe image noise, or inadequate contrast enhancement. These factors can hinder the accurate assessment of coronary arteries, particularly in heavily calcified vessels. Our study highlights the importance of modern scanner technologies and rigorous imaging protocols in minimizing diagnostic errors.

### 4.2. Integration into the Literature

In contrast to the present study, previous studies focusing on pre-TAVI-CT primarily assessed the overall diagnostic performance to detect obstructive disease compared to ICA; they did not specifically address the inaccurate interpretation of coronary artery stenosis with this modality. For example, van den Boogert and colleagues [9] investigated the ability of CT angiography to assess the presence and severity of CCS in TAVI candidates. As recommended by the guidelines on the management of patients with valvular heart disease [3], a diameter stenosis threshold of ≥70% in the proximal vessel segments was defined as significant CCS. In this setting, the diagnostic yield of CT angiography evaluation in patients prior to TAVI theoretically reduced the need for subsequent ICA in 70%.

In the most recent meta-analysis, Becker et al. [10] investigated the value of pre-TAVI CT angiography with and without CT-derived FFR for ruling out relevant CAD. Their findings likewise suggest that pre-TAVI-CT can accurately rule out obstructive stenosis in patients prior to TAVI. However, a detailed appraisal of the high number of FP diagnoses and causal factors is lacking. In general, the identification of specific clinical and imaging predictors associated with diagnostic inaccuracies in coronary artery stenosis detection using pre-TAVI-CT is underexplored in the current literature. In contrast to the meta-analysis by Becker et al. [10], our work is centered on the factors associated with FP and FN coronary stenosis interpretation. By systematically analyzing patient characteristics, vessel-specific parameters, and CT image quality, this work offers novel aspects and provides a better understanding of the variables influencing the diagnostic reliability of preprocedural CT angiography in patients prior to TAVI and therefore contributes to the current literature. Our approach is targeted at a gap in knowledge and advises a more individualized diagnostic pathway for candidates being evaluated for TAVI.

Using an approach similar to ours, Yan et al. [16] sought to identify clinical and readily discernible imaging results predicting the inaccurate diagnosis of obstructive CAD by CT angiography. As expected, coronary artery calcification was associated with FP stenosis assessment in both studies, although its discriminatory value was not independent after the adjustment for other vessel-level factors like those used in the present study. Nevertheless, this finding aligns with the existing literature, demonstrating that the presence of coronary artery calcium is associated with reduced specificity and negative predictive values [17,18]. At the same time, the risk of the under-recognition of non-calcified plaques by CT angiography has previously been established [16,19], a finding that may have contributed to an increased risk of false coronary artery assessments in younger patients here. Yan et al. [16] identified independent predictors at the segment level, including localized tortuosity, luminal diameter of an affected segment, and juxta-arterial vein conspicuity. These parameters were not assessed in our study. We chose vessel-level rather than segment-level analysis, because localized stenoses were scarce, and in cases with CCS in these elderly patients with AS, a more diffuse disease was present.

Notably, there are critical differences between the study populations and methodologies that likely account for divergent findings. All of our patients suffered from severe symptomatic AS and presented a median overall coronary artery calcium score of >400, and nearly half of them had atrial fibrillation. At the same time, these were important exclusion criteria for the population of patients studied by Yan et al. [16], which stemmed from the prospective multicenter trial CORE-64 [20]. In addition, our patients were significantly older (82 ± 5 vs. 59 ± 10 years). With respect the reference standard, our study defined ≥ 70% luminal diameter stenosis by ICA or fractional flow reserve ≤ 0.80 as obstructive CAD, whereas the previous study used ≥50% coronary stenosis as the criterion. These differences highlight the unique characteristics of our study population and methodology, underscoring the need for tailored diagnostic approaches in this high-risk group of patients.

The quality of pre-TAVI-CT imaging plays a pivotal role in the accurate detection of coronary artery stenosis, as suboptimal image quality can significantly impair diagnostic performance [21]. Common issues such as high body mass index, breathing artifacts, increased image noise, and poor contrast opacification can severely impair the interpretability of coronary artery images, leading to FP or FN results [22]. These challenges are pronounced in elderly patients with degenerative AS, where comorbidities such as atrial fibrillation and elevated heart rates complicate CT image acquisition. In this context, the advanced preparation of patients with severe AS for pre-TAVI-CT, including administering beta-blockers or nitroglycerine, was reported to be safe and could be considered [23].

### 4.3. Clinical Implications and Future Directions

As previously shown, coronary artery information from pre-TAVI-CT is worthwhile to be utilized [7]. As a next step, the identification of the predictors of pre-TAVI-CT misdiagnoses shown in the present work provides practical guidance for clinicians. For patients with risk factors such as a younger age or atrial fibrillation, as suggested by the univariate analysis, a more cautious interpretation of pre-TAVI-CT is recommended. Investing in higher-quality imaging equipment or optimizing scanning protocols could significantly enhance diagnostic performance. These findings are likely to become even more relevant due to the steadily increasing proportion of younger and fitter patients with lower overall surgical risk being considered for TAVI. This will at the same time increase the value of pre-TAVI-CT for the exclusion of relevant coronary artery stenosis.

Our findings suggest a potential role for individualized diagnostic pathways. For patients who are at high risk of pre-TAVI-CT inaccuracies, the involvement of noninvasive stress tests or invasive clarification via ICA potentially involving further work-up with FFR may be necessary to ensure the accurate identification of clinically significant CCS.

Further research is warranted to validate our findings in prospective, multicenter studies and to reassess the utility of predictive models incorporating the factors suggested by the present work. Exploring the yield of pre-TAVI-CT utilizing integrated artificial intelligence for real-time image quality assessment and risk stratification could improve diagnostic workflows [24].

### 4.4. Study Limitations

This study should be viewed in light of its limitations. First, because of the study design, inherent selection biases need to be considered. Randomized controlled trials are warranted for validation purposes. Second, the total number of patients included and, in particular, the subset of those classified as FN was limited. Therefore, we refrained from performing subgroup analyses that would not have been valid. Our exploratory analysis should be regarded as hypothesis-generating and we recommend that our results be confirmed in larger patient populations. Third, this was not an all-comers population, and the application of exclusion criteria limits the generalizability of our results. However, a similar framework setting could ensure a higher diagnostic value of pre-TAVI-CT, as suggested previously [7,13]. Fourth, the results of FFR and resting pressure ratios are to be interpreted with caution, although their utility in patients with severe AS was reported [25]. Generally, FFR in the presence of AS may be FN and underestimate the hemodynamic relevance of coronary artery stenosis.

### 4.5. Conclusions

Clinical and imaging parameters can serve as predictors of the poorer accuracy of CAD assessment by pre-TAVI-CT in comparison with ICA. Based on the results of our retrospective study, a younger patient age and insufficient image quality were independently associated with a misdiagnosis. Herewith, this work adds novelty and provides a better understanding of the variables adversely influencing diagnostic reliability. Knowledge of these predictors may therefore aid in an individualized and at the same time more efficient coronary artery image interpretation by pre-TAVI-CT beyond guideline recommendations.

## Figures and Tables

**Table 1:** Clinical and imaging characteristics according to patient-level diagnostic discordance between CT angiography and the invasive reference standard.

	Diagnostic Concordance of CT Angiography (Accuracy; TP and TN) *n* = 158	Diagnostic Discordance of CT Angiography (Inaccuracy; FP and FN) *n* = 34	*p*-Value
*Patient characteristics*			
Sex (female)	103 (65.2)	19 (55.9)	0.31
Age, years	82.4 [80.1–85.5]	79.8 [76.8–84.2]	0.01
Body mass index, kg/m^2^	26.3 [24.0–30.4]	27.5 [24.9–32.8]	0.13
Diabetes	40 (25.3)	11 (32.4)	0.39
Hypertension	148 (93.7)	34 (100)	0.13
Dyslipidemia	41 (25.9)	7 (20.6)	0.52
Smoking	18 (11.4)	5 (14.7)	0.59
Family history of CAD	8 (5.1)	1 (2.9)	0.58
Atrial fibrillation	62 (39.2)	19 (55.9)	0.07
NYHA class	3 [3–3]	3 [2–3]	0.64
eGFR, mL/min/1.73 m^2^	70.0 [52.0–84.8]	67.5 [51.3–83.8]	0.64
*Results from echocardiography*			
LVEF, %	62.0 [55.0–65.0]	60.0 [60.0–65.0]	0.22
AVA, cm^2^	0.7 [0.5–0.9]	0.7 [0.5–0.8]	0.77
*CT imaging parameters*			
CT system (not capable of single-heartbeat acquisition)	88 (55.7)	26 (76.5)	0.03
Heart rate during CT, bpm	71.0 [63.0–80.0]	77.5 [66.5–80.0]	0.12
Image quality (1 = non-diagnostic to 5 = excellent)	3.5 [3–4]	3 [1–3]	<0.01
Agatston score, HU	468.5 [147.2–1202.5]	679.1 [242.2–1179.3]	0.69

Values denote n (%) or median [interquartile range]. Abbreviations: AVA, aortic valve area; CAD, coronary artery disease; eGFR, estimated glomerular filtration rate; FP, false-positive; FN, false-negative; LVEF, left ventricular ejection fraction; NYHA, New York Heart Association; TP, true-positive; TN, true-negative.

**Table 2:** Vessel and lesion characteristics on CT according to vessel-level diagnostic discordance between CT angiography and the invasive reference standard.

	Diagnostic Concordance of CT Angiography (Accuracy; TP and TN) *n* = 506	Diagnostic Discordance of CT Angiography (Inaccuracy; FP and FN) *n* = 70	*p*-Value
Vessel-specific signal-to-noise ratio	15.3 [12.0–18.9]	13.7 [10.2–19.9]	0.14
Lesion location * Left main artery LAD LCx RCA	9 (1.8) 168 (33.4) 176 (34.8) 162 (32.0)	1 (1.4) 24 (34.3) 16 (22.9) 30 (42.9)	0.83 0.81 0.04 0.07
Vessel-specific Agatston score, HU	95.8 [11.9–290.9]	200.2 [54.2–573.2]	<0.01
Plaque composition (calcified vs. non-calcified) **	366/397 (92.2) vs. 31/397 (7.8) **	60/64 (93.8) vs. 4/64 (6.2) **	0.66

Values denote n (%) or median [interquartile range]. Abbreviations: FP, false-positive; FN, false-negative; LAD, left anterior descending coronary artery; LCx, left circumflex coronary artery; RCA, right coronary artery; TP, true-positive; TN, true-negative. * The diagonal branches were assigned to the LAD, the ramus posterolateralis dexter was assigned to the RCA and the obtuse marginal branches, and the ramus intermedius and the ramus posterolaterialis sinister were assigned to the LCx. ** In 115/576 vessels, there was no lesion, or the lesion morphology was not assessable, e.g., due to artifacts.

**Table 3:** Multivariable logistic regression analysis based on univariate analysis of patient and imaging criteria at the patient level using misdiagnosis of CT angiography according to the invasive reference standard.

	Overall Misdiagnosis *n* = 34	
	Odds Ratio (95% Confidence Interval)	*p*-Value
Age, per year	0.87 (0.80–0.94)	<0.01
Atrial fibrillation	1.81 (0.79–4.16)	0.16
CT system (not capable of single-heartbeat acquisition)	2.51 (0.96–6.56)	0.06
CT image quality (1 = non-diagnostic–5 = excellent)	0.60 (0.41–0.89)	<0.01

**Table 4:** Multivariable logistic regression analysis based on univariate analysis of vessel and lesion criteria at the vessel level, using misdiagnosis of CT angiography according to the invasive reference standard.

	Overall Misdiagnosis *n* = 70	
	Odds Ratio (95% Confidence Interval)	*p*-Value
LCx	0.70 (0.35–1.37)	0.30
RCA	1.28 (0.71–1.37)	0.40
Vessel-specific Agatston score, HU	1.00 (1.00–1.00)	0.11

Abbreviations: LCx, left circumflex coronary artery; RCA, right coronary artery.

## Data Availability

The data presented in this study are available on request from the corresponding author. The data are not publicly available due to privacy restrictions.

## Supplementary Materials

The following supporting information can be downloaded at: https://www.mdpi.com/article/10.3390/diagnostics15060771/s1, Table S1: Clinical and imaging characteristics according to patient-level diagnostic discordance (separately shown for false-positive and false-negative assessment) between CT angiography and the invasive reference standard; Table S2: Vessel and lesion characteristics on CT according to vessel-level diagnostic discordance (separately shown for false-positive and false-negative assessment) between CT angiography and the invasive reference standard; Table S3: Multivariable logistic regression analysis based on univariate analysis of patient and imaging criteria at the patient level using misdiagnosis of CT angiography according to the invasive reference standard; Table S4: Multivariable logistic regression analysis based on univariate analysis of vessel and lesion criteria at the vessel level, using misdiagnosis of CT angiography according to the invasive reference standard.
