# Supplementary material for: Predictors of Diagnostic Inaccuracy of Detecting Coronary Artery Stenosis by Preprocedural CT Angiography in Patients Prior to Transcatheter Aortic Valve Implantation
Source: Diagnostics (Basel). 2025 Mar 19;15(6):771. doi: 10.3390/diagnostics15060771 (PMC11941401; doi:10.3390/diagnostics15060771)
Supplement: Supplementary file 1 [file diagnostics-15-00771-s001.zip › diagnostics-3486826-supplementary.pdf]

Supplementary Table S1: Clinical and imaging characteristics according to patient-level diagnostic discordance (separately shown for false-positive and false-negative assessment) between CT angiography and the invasive reference standard.

	Diagnostic Concordance of CT angiography (Accuracy; TP and TN) n=158	FP Assessment by CT angiography (Inaccuracy; FP) n=30	FN Assessment by CT angiography (Inaccuracy; FN) n=4
Patient characteristics			
Sex (female)	103 (65.2)	17 (56.7)	2 (50.0)
Age, years	82.4 [80.1-85.5]	80.6 [77.1-85.0]	78.3 [72.9-79.9.2]
Body mass index, kg/m²	26.3 [24.0-30.4]	27.4 [24.6-32.7]	28.9 [27.0-31.0]
Diabetes	40 (25.3)	9 (30.0)	2 (50.0)
Hypertension	148 (93.7)	30 (100)	4 (100)
Dyslipidemia	41 (25.9)	6 (20.0)	1 (25.0)
Smoking	18 (11.4)	4 (13.3)	1 (25.0)
Family history of CAD	8 (5.1)	1 (3.3)	0 (0)
Atrial fibrillation	62 (39.2)	19 (55.9)	0 (0)
NYHA class	3 [3-3]	3 [2-3]	3 [2.8-3.3]
eGFR, ml/min/1.73 m²	70.0 [52.0-84.8]	64.0 [51.0-80.8]	76.5 [72.0-81.3]
Results from echocardiography			
LVEF, %	62.0 [55.0-65.0]	65.0 [60.0-65.0]	62.5 [60.0-65.0]
AVA, cm²	0.7 [0.5-0.9]	0.7 [0.5-0.8]	0.8 [0.7-0.8]
CT imaging parameters			
CT system (not capable of single-heartbeat acquisition)	88 (55.7)	23 (76.7)	3 (75.0)
Heart rate during CT, bpm	71.0 [63.0-80.0]	77.0 [66.5-80.0]	77.5 [72.8-82.5]
Image quality (1=non-diagnostic to 5=excellent)	3,5 [3-4]	2 [1-3]	3 [3-3.5]
Agatston score, HU	468.5 [147.2-1202.5]	699.6 [247.2-1221.9]	579.4 [345.4-778.7]

Values denote n (%) or median [interquartile range].

Abbreviations: AVA, aortic valve area; CAD, coronary artery disease; eGFR, estimated glomerular filtration rate; FP, false-positive; FN, false-negative; LVEF, left ventricular ejection fraction; NYHA, New York Heart Association; TP, true-positive; TN, true-negative.

Supplementary Table S2: Vessel and lesion characteristics on CT according to vessel-level diagnostic discordance (separately shown for false-positive and false-negative assessment) between CT angiography and the invasive reference standard.

	Diagnostic Concordance of CT angiography (Accuracy; TP and TN) n=506	FP Assessment by CT angiography (Inaccuracy; FP) n=60	FN Assessment by CT angiography (Inaccuracy; FN) n=10
Vessel-specific signal-to-noise ratio	15.3 [12.0-18.9]	13.7 [9.5-17.6]	18.5 [11.5-22.5]
Lesion location*			
• Left main artery	9 (1.8)	1 (1.7)	0 (0)
• LAD	168 (33.4)	21 (35.0)	3 (30.0)
• LCx	176 (34.8)	14 (23.3)	2 (20.0)
• RCA	162 (32.0)	25 (41.7)	5 (50.0)
Vessel-specific Agatston score, HU	95.8 [11.9-290.9]	198.2 [50.7-669.0]	208.9 [54.2-573.2]
Plaque composition (calcified vs. non-calcified)**	366/397 (92.2) vs. 31/397 (7.8)**	51/54 (94.4) vs. 3/54 (5.6)**	9/10 (90.0) vs. 1/10 (10.0)**

Values denote n (%) or median [interquartile range].
Abbreviations: FP, false-positive; FN, false-negative; LAD, left anterior descending coronary artery; LCx, left circumflex coronary artery; RCA, right coronary artery; TP, true-positive; TN, true-negative.
* The diagonal branches were assigned to the LAD, the ramus posterolateralis dexter was assigned to the RCA and the obtuse marginal branches, ramus intermedius and the ramus posterolaterialis sinister were assigned to the LCx.
** In 115/576 vessels, there was no lesion or the lesion morphology was not assessable, e.g. due to artifacts.

**Supplementary Table S3:** Multivariable logistic regression analysis based on univariate analysis of patient and imaging criteria at the patient level using misdiagnosis of CT angiography according to the invasive reference standard.		
	**Overall misdiagnosis n=34**	
	**Odds ratio (95% confidence interval)**	**p-value**
Age, per year	0.87 (0.80-0.94)	<0.01
Body mass index, kg/m²	1.04 (0.96-1.13)	0.34
Hypertension	$683*10^{6}$ (n.a.)	0.99
Atrial fibrillation	1.81 (0.79-4.16)	0.41
Heart rate during CT, bpm	1.01 (0.97-1.04)	0.74
CT system (not capable of single-heartbeat acquisition)	2.51 (0.96-6.56)	0.06
CT image quality (1=non-diagnostic – 5=excellent)	0.60 (0.41-0.89)	<0.01

**Supplementary Table S4:** Multivariable logistic regression analysis based on univariate analysis of vessel and lesion criteria at the vessel level, using misdiagnosis of CT angiography according to the invasive reference standard.		
	**Overall misdiagnosis n=70**	
	**Odds ratio (95% confidence interval)**	**p-value**
Vessel-specific signal-to-noise ratio	1.00 (0.96-1.04)	0.97
LCx	0.70 (0.35-1.37)	0.30
RCA	1.28 (0.71-1.37)	0.40
Vessel-specific Agatston score, HU	1.00 (1.00-1.00)	0.11
